# In a trauma experimental pig model prothrombin complex concentrates and a specific antidote (idarucizumab) are effective to reverse the anticoagulant effects of dabigatran

**DOI:** 10.1186/cc13308

**Published:** 2014-03-17

**Authors:** O Grottke, J Van Ryn, R Rossaint

**Affiliations:** 1 RWTH Aachen, Germany; 2 Boehringer Ingelheim, Biberach, Germany

## Introduction

Strategies to reverse the anticoagulant effects of the new oral anticoagulants in severely bleeding patients remain challenging and conflicting results have been presented regarding the efficacy of PCCs to alter dabigatran-induced coagulopathy. Thus, this study assessed the ability of PCC, activated PCC (aPCC), recombinant FVII (rFVIIa) and idarucizumab to reverse the anticoagulant effects of dabigatran in a porcine model of trauma.

## Methods

Studies were performed in five pigs. Dabigatran etexilate (DE) was given orally for 3 days (30 mg/kg bid) and on the 4th day dabigatran was infused 90 minutes (30 minutes: 0.77 mg/kg/hour; 60 minutes 0.52 mg/kg/ minute) prior to blunt liver injury. Blood samples were taken before and after dabigatran infusion and 60 minutes post injury. Two doses of PCC (30, 60 IU/kg), aPCC (30, 60 IU/kg), rVIIa (90, 180 μg/kg) and idarucizumab (30, 60 mg/kg) were added to blood samples *ex vivo. *Coagulation was assessed by thromboelastometry, PT and diluted TT. One-way analysis of variance with the Dunnett *post-hoc *test for multiple comparisons was used for statistical analysis. Data are presented as mean ± SD.

## Results

Oral DE prolonged clot formation (CFT: 159 ± 39 seconds) and PT (27 ± 9 seconds). Following the 90-minute infusion of dabigatran, the mean plasma levels of dabigatran increased to 1,423 ± 432 ng/ ml. This supra-therapeutic level was associated with a further prolongation of PT, aPTT, and the EXTEM variables CT and CFT. These changes in coagulation parameters were compounded by blood loss following trauma (total blood loss at 60 minutes: 1,978 ± 265 ml). Sixty minutes after trauma, four out of five animals had no measurable clot formation (EXTEM CFT ≥4,000 seconds) and clot strength (EXTEM MCF) had reduced to 11 ± 7 mm. Both PCCs and aDabi-Fab, but not rFVIIa, reversed the effects of dabigatran on thromboelastometry parameters (clotting time and clot formation time) and PT at all time points. In contrast, aPTT was only normalised by idarucizumab. Plasma concentrations of dabigatran remained elevated after PCC therapy, but were not measureable after idarucizumab.

## Conclusion

Both PCC and aPCC are effective in reducing coagulopathy in a porcine trauma model with dabigatran anticoagulation. The *ex vivo *addition of idarucizumab fully corrected all coagulation measures and significantly decreased plasma concentrations of dabigatran. No significant effects on haemostasis were observed after the application of rFVIIa.

